# Towards Commercial Production of Sponge Medicines

**DOI:** 10.3390/md7040787

**Published:** 2009-12-02

**Authors:** Marieke Koopmans, Dirk Martens, Rene H. Wijffels

**Affiliations:** Bioprocess Engineering Group, Department of Agrotechnology and Food Sciences, Wageningen University, P.O. Box 8129, 6700 EV Wageningen, The Netherlands; E-Mail: Marieke.Koopmans@wur.nl (M.K.)

**Keywords:** bioactive metabolite, biosynthetic pathway, sponge medicine, sponge culture, cell culture

## Abstract

Sponges can provide potential drugs against many major world-wide occurring diseases. Despite the high potential of sponge derived drugs no sustainable production method has been developed. Thus far it is not fully understood why, when, where and how these metabolites are produced in sponges. For the near future sea-based sponge culture seems to be the best production method. However, for controlled production in a defined system it is better to develop *in vitro* production methods, like *in vitro* sponge culture or even better sponge cell culture, culture methods for symbionts or the transfer of production routes into another host. We still have insufficient information about the background of metabolite production in sponges. Before production methods are developed we should first focus on factors that can induce metabolite production. This could be done in the natural habitat by studying the relation between stress factors (such as predation) and the production of bioactive metabolites. The location of production within the sponge should be identified in order to choose between sponge cell culture and symbiont culture. Alternatively the biosynthetic pathways could be introduced into hosts that can be cultured. For this the biosynthetic pathway of metabolite production should be unraveled, as well as the genes involved. This review discusses the current state of sponge metabolite production and the steps that need to be taken to develop commercial production techniques. The different possible production techniques are also discussed.

## 1. Introduction

Marine sponges are a ‘gold mine’ with respect to the diversity of their secondary metabolites discovered during the past fifty years. Sponges can provide potential drugs against many major world-wide occurring diseases. Of the 18,000 marine natural products described, over 30% are from sponges and of the antitumor natural product patent registrations in recent years over 75% are from sponges [1,2]. An overview of marine natural products and their status in clinical trials can be found at: www.marinebiotech.org/pipeline.html. Many of these products originate from sponges.

Sponges (phylum Porifera) appear to be very stable, long-lived animals, with growth rates that vary enormously between different groups. They are multi-cellular filter-feeding invertebrates living attached to a substratum in mostly marine, but also freshwater habitats. Sponges do not possess true tissue, but have different cell types with different functions, which together carry out normal body functions. To provide the sponges with food and oxygen and to excrete waste products a large amount of seawater is filtered. Many sponges have a symbiosis with micro-organisms. Symbionts include archaea, bacteria, fungi, cyanobacteria, and microalgae. Photosynthetic symbionts provide the sponge with photosynthetic nutrients, which they do not obtain by their own filter-feeding activities.

For the development of a sustainable production method of sponge bioactive metabolites, more knowledge is necessary about the biology and the needs of the sponge in nature for both growth and metabolite production. There has been a lot of attention on the discovery of new bioactive metabolites. To develop these bioactive metabolites into medicines they need to be tested in clinical trials, for which substantial amounts of compounds are needed. The concentration of bioactive compounds in sponge tissue can be very low. For example, *Lissodendorix* sp. contains concentrations of about 400 μg/kg of halichondrin B. This means that there is a need for a sustainable production method. To develop a sustainable production technique it must be known whether the sponge or its symbionts or both are responsible for the production of the bioactive compound and in the case the sponge is the producer, which cells are responsible for the production. Because of the low concentrations inside the sponge the amount of sponge biomass needed will be very high [3,4]. To be able to increase the production per sponge, more knowledge is needed on the biosynthetic pathways and their regulation, which includes factors that induce production of the metabolites [5].

There are several possible strategies to produce sponge bioactive compounds. Wild harvest is ecologically undesirable, due to the large amounts of sponge biomass necessary for commercial applications [6]. We believe that potential strategies to produce bioactive compounds from sponges include sponge or sponge cell cultures and genetic modification approaches in which large gene fragments responsible for production of the bioactive compounds are identified and transferred into a suitable host. So far, none of the approaches resulted in applicable technologies for production of sponge bioactive compounds despite significant progress. In this paper we will discuss the two major bottlenecks for sponge metabolite production, namely: Understanding metabolite production in the sponge, and choosing and improving culture systems. We propose a strategy how to develop a sponge metabolite production process in the following order:

1. To understand metabolite production following steps are needed:
  - Identification of induction factors of metabolite production.
  - Identifying biosynthetic pathways of secondary metabolites.
  - Identification of the location of bioactive compound production.
2. Choosing and improving one of the following culture systems:
  - Whole sponge culture.
  - Sponge cell culture.
  - Symbiont culture.
  - Genetic modification.

## 2. Understanding Metabolite Production in the Sponge

### 2.1. Induction of Sponge Metabolite Production

**Figure 1.**
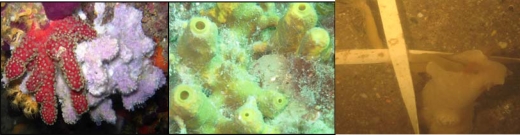
Sponges competing: (A) *Dysidea avara* (right) competing for space with a soft leather coral, (B) *Aplysina aerophoba* fouled with hydrozoa, (C) *Haliclona oculata* predated by a nudibranch.

It is generally assumed that sponges produce secondary metabolites because they have to compete for space with other organisms, they have to prevent fouling by other organisms and they have to keep predators away [7] (Figure 1). The highest frequency of toxic or deterrent metabolites is found in high competing environments. Bioactive compounds are for example often discovered in coral reefs with an enormous biodiversity. Furthermore, sponges found in exposed areas that are vulnerable to fish predators are usually more toxic than those growing unexposed [8]. For example, chemical deterrence of fish predators was found significantly higher for extracts obtained from the tropical sponge community as compared to the temperate community where less predation occurs [9]. Predation pressure thus increases investment into chemical defense and sponges have evolved in higher or lower bioactive compound producing specimen due to possible predation.

Sponge bioactive metabolite concentrations were found to be variable corresponding to several environmental factors. The season and variation in temperature, location and illumination all have significant effect on bioactive metabolite concentration. A positive correlation between temperature and metabolite concentration was found for salicylihalmide A in *Haliclona* sp. [10], and mycalamide A in *Mycale hentscheli* [11]. *Crambe crambe* from the Mediterranean appears to be less toxic in well illuminated habitats compared to shaded habitats [12]. Also for other invertebrates variations were found. For example, the bioactive compound ascididemin from the ascidian *Cystodytes* sp. shows a seasonal trend and a positive correlation with temperature [13].

The sponge *Dysidea avara* (Figure 1) competes for space with a leather coral. It is expected that under such circumstances the sponge produces more bioactive compounds than in absence of space competing organisms. There are only a limited number of such cases that have been studied, but they all support this hypothesis [14,15]. Several studies have shown that sponges and sponge extracts are deterrent towards predators like fishes [16–19]. It is often suggested that sponges are induced to produce more bioactive compounds when predated [7]. In terrestrial plants this mechanism has been well studied. The most common inducer of secondary metabolites in plants is herbivory by insects [20]. Also, in gorgonians it has been shown that the natural product content could be increased significantly in the presence of predator snails [21].

There are several examples that support the theory of possible induction of metabolite production due to stress factors. Transplantation, infection and different diets showed to induce metabolite production. Individuals transplanted from depth to near surface substantially increased the production of the natural product diterpenes in harvested sponge tissue of *Rhopaloeides odorabile* [22]. *Aplysina aerophoba* increased aplysinimine production in regions of the sponge close to infected sponge tissue, suggesting a defense response to the denser microbial community in the diseased part of the sponge [23]. Stevensine (an alkaloid metabolite) levels were significantly elevated in the experimental group of sponge explants cultured *in vitro* that were given a mixed diet of food particles at three times the natural environment particle concentration compared to sponges fed normal particle concentration [24].

Another possibility to induce metabolite production is called activated chemical defense, which is induced by wounding. The probable ecological relevance of this mechanism is protection of the damaged sponge tissue from invasion of bacterial pathogens [25]. Wound activation of protoxins results in a pronounced increase of fish deterrent activity of *A. aerophoba* [26]. In *Aplysina* it was shown that wound-activated chemical defense occurs most likely by enzymatic conversion [27]. Due to wounding, brominated isoxazoline alkaloids were converted into the monocyclic nitrogenous compounds aeroplysinin-1 and dienone by enzymatic cleavage [25]. Wound regeneration was found to be slower in sponges with chemical defense than in sponges without chemical defense [28]. The difference in regeneration rate is suggested to be the result of two different strategies where the sponge either invests in a high regeneration rate or in producing compounds for chemical defense.

Despite the differences found in bioactive metabolite concentrations at different environmental circumstances, for most sponges it is still unknown which factors induce production of bioactive compounds. As sponges react to predation, wounding and stress in general, there are probably specific inducers that trigger these responses. No studies have been performed to find which specific compounds induce secondary metabolite production inside the sponge. Finding such inducers would be of great value, since it would allow the induction of chemical defense and the associated metabolite production without the negative effects of, for example, wounding. If, for example, studies *in situ* reveal that production is induced due to the presence of predators. Extracts of the predator can be analyzed and gradually purified and tested on the sponges with the goal to identify the bioactive compound inducing chemical defense.

### 2.2. Biosynthesis of Secondary Metabolites

Inducible production of bioactive compounds can also be helpful in identifying the genes involved. Information on the genes involved in metabolite production can then be obtained by comparing the gene expression before and after inducing bioactive metabolite production. A sequenced genome or *a priori* knowledge of enzymes involved greatly facilitates this approach.

The complex interaction between sponges and symbionts is one of the reasons that relatively little genome research is done on sponges. Technically, it is difficult to separate the DNA of the sponge from that of the other organisms. Recently, the first sponge genome project was launched by the University of Queensland, Australia. The only sponge of which the genome is sequenced and published is *Amphimedon queenslandica* (www.compagen.org). In order to make faster progress in drug development a method is necessary with which it is possible to screen complex genetic material. Metagenomics has developed to be valuable as a tool for studying complex communities [29].

Sponges can be seen as complex communities due to the presence of many symbionts. Developments in metagenomics have provided new insights in sponge metabolite production [30,31]. It can be used to find if certain pathways are of bacterial or sponge origin. For example, polyketide synthase (PKS) enzymes are involved in the synthesis of many natural products [32] and were found to be of bacterial origin inside several sponges [31,33]. Identifying gene fragments involved in bioactive metabolite production becomes more difficult when more complex metazoan genomes are studied for isolation of specific biosynthetic genes [33]. A targeted approach should be used to begin this complex study. Many marine natural products are terpenes [34] and thus a good start to investigate gene expression in sponges would be to focus on the mevalonate pathway, which is a known common terpene biosynthesis pathway [5].

Another technique to get more insight in sponge metabolism is to study the metabolome, *i.e.*, the complete set of metabolites. Techniques to study the metabolome have developed fast in the recent years [35]. The metabolome is the final product of gene expression in a cell and thus represents the interaction of all biochemical processes. Metabolome studies in sponges on bioactive compounds have not been done yet, but it is done in sponges for fatty acid (FA) metabolism. Biosynthesis of fatty acids has been studied in different sponges by labeling techniques [36,37]. ^14^C-label was used by Hahn *et al*. to elucidate the biosynthesis of two long chain ‘demospongic acids’ in the sponge *Microciona prolifera*. Synthesis of these fatty acids was found to be done by elongating exogenous short chain FAs. Carbon isotopic labeling can be used to study intermediates of different compounds, or labeled intermediates can be fed to study further conversion pathways [38]. Multigene expression analysis in combination with metabolic analysis can be used to study production of bioactive compounds under stress conditions. This enables monitoring of expression levels of genes involved in the biosynthetic route of the bioactive compound.

### 2.3. Location of Secondary Metabolite Production in the Sponge

To be able to choose a production system it is necessary to know where the metabolites are produced in the sponge. Very often it is not known whether the sponge or the sponge symbiont is responsible for the production of the bioactive compound. Even in cases this is known, it is not known if the sponge-symbiont relation is important for the production of the bioactive compound. It is often suggested, but has never been demonstrated, that in case the bioactive compound is produced by the sponge cell, the precursor for that compound might have been produced by an associated microorganism. Even in cases the compound is present in the sponge cell it may have been produced by a symbiont and stored in the sponge cell. It is often suggested that sponge symbionts are so tightly coupled that the microbial genome size is reduced, making the symbiont for nutrition dependent of the sponge or even that the genomes of sponge and symbiont are integrated [39–41].

In different studies it was shown that sponge bioactive compounds are stored in specific parts of the sponge, such as the outer layer, which is most exposed to predators. The secondary metabolite desacetylscalaradial was found in higher concentrations in the tips than in the base of the branching sponge *Cacospongia* sp. [42]. In *Rhopaloeides odorabile*, diterpene concentrations were found highest in the surface tissue [22]. Similar results were found for *Ectoplasia ferox* as larger concentrations of triterpene glycosides were found in the top layer of the sponge tissue [43]. However, the same article presents the opposite for *Erylus formosus*, where higher concentrations of formoside were found in the inner part of the sponge. Thus several studies support that highest concentrations are produced in the most vulnerable parts of the sponge.

**Figure 2.**
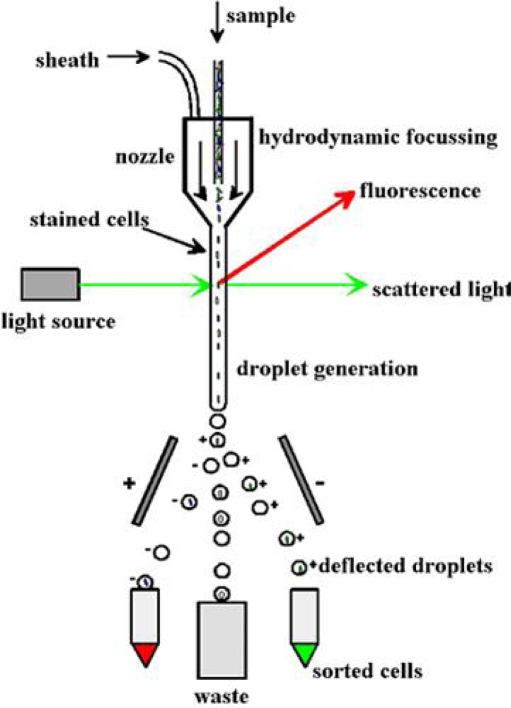
Flow sorting using flow cytometry. Cells can be charged based on difference in fluorescence or size (http://missinglink.ucsf.edu/lm/molecularmethods/flow.htm).

Several methods have been used to identify the location of the bioactive compound in the different cell types of the sponge and its symbionts. For this the different cell fractions from sponges are isolated by flow sorting [44] (Figure 2) and Ficoll or Percoll density gradient centrifugation [45,46]. These cell fractions can then be analyzed for bioactive metabolites and intermediates of the biosynthetic route. An example of a bioactive compound produced by the sponge is stevensine from *Axinella corrugata*. Stevensine was also produced in a primary sponge cell culture [47]. Unson and Faulkner [44] found that cyanobacteria are responsible for the production of chlorinated metabolites, however the sequiterpenoids were only found in the sponge cells. In the Haplosclerid sponge *Haliclona* sp. the cytotoxic alkaloids were located in the sponge cells [45]. This is also the case for avarol from *Dysidea avara*, which is located in the choanocytes [46].

In some cases, the symbionts and not the sponge cells are the likely source of the secondary metabolites of interest [48,49]. For example, the polybrominated biphenyl ether antibiotics isolated from the sponge *Dysidea herbacea* are produced by the endosymbiotic cyanobacterium *Oscillatoria spongeliae* [44]. Manzamine in the sponge *Acanthostrongylophora* is produced by the associated microorganism from *Micromonospora* sp [50]. Another example is the production of dysiherbaine in the endosymbiotic cyanobacterial cells of the genus *Synechocystis* in the sponge *Lendenfeldia chondrodes* [51]. Fungi associated with marine sponges are also known to produce many bioactive agents [52]. Work on isolation and cultivation of sponge symbionts and the nature of symbiotic relationships have been reviewed elsewhere [48,53].

Cell types that can be isolated to study the location of metabolite production are bacteria, microalgae and different types of sponge cells in which presence of bioactive compounds is expected. Sponge cell types of interest in this respect are the archeaocytes, choanocytes and pinacocytes. It is mostly suggested that archeaocytes are responsible for the production of secondary metabolites. Since one of the reasons to make bioactive compounds is protection against predators, one would expect these compounds to be present in the pinacocytes, which form the outer layer of the sponge. However, so far it is not shown that pinacocytes contain bioactive compounds. When compounds are found in specific cells it can still be that intermediates or precursors may have been produced by associated microorganisms or other cells. The debate about this is mainly based on speculation and we have to conclude that at present it is unknown in which cell types the bioactive compounds and their intermediates are produced.

## 3. Culture Systems

### 3.1. Sponge Culture

**Figure 3.**
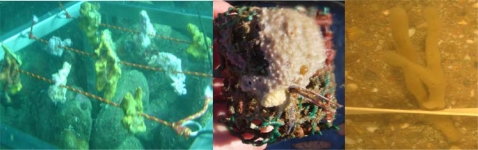
Different attachment forms for sea-based culture. Sponges on (A) threads, (B) in cages, (C) on tiles, attached using a plastic band, where sponges attach to the tile themselves within one month.

Sipkema *et al*. [4] concluded that whole sponge culture is the most promising option for large scale production of higher concentrated metabolites like avarol, which is produced up to 3 g per kg sponge wet weight [54] in *Dysidea avara*. Especially for faster growing sponges containing large amounts of metabolites whole sponge culture is potentially interesting. It was determined that sea-based culture is economically cheaper than land-based culture [4]. The major drawback of culturing sponges is that many sponges were found to be slow or variable growing organisms both in the sea [55–60] as well as in the laboratory [61–67]. Studies have demonstrated that production of metabolites in cultured sponges was sometimes lower [57], but on the other side also higher production rates were found [11,68] than in sponges harvested from the sea. Apart from the costs, sea-based culture is less desirable than land-based culture as the conditions cannot be controlled in the sea, and the sponges are vulnerable to diseases and parasites. Several attempts have been made to culture sponges on land. Most of the research focused on food requirements, which is thought to be the key to success [69]. Sponge cultures in the sea can be done using different structures for attaching sponges (Figure 3), and harvesting could be done partially thus leaving explants behind to re-grow. Sea-based cultivation is still the method where the largest growth rates have been obtained. Apparently, the artificial environment we construct for the sponges still cannot replace the complexity of the sea. A good alternative for this is to culture sponges on land but still use natural seawater flowing through the aquaria containing the sponges. Despite several growth studies still very limited understanding is present about the exact needs of sponges to improve growth. Therefore, before sponge culture systems can be realized more understanding is necessary about food, attachment surfaces and other requirements that stimulate sponge growth.

### 3.2. Sponge Cell Culture

**Figure 4.**
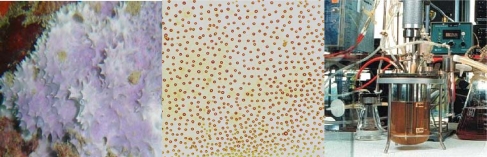
Development of sponge cell-lines. (A) Dysidea avara (B) cells isolated from Dysidea avara (C) bioreactor to culture animal cells.

In the cases that the sponge cells are responsible for the bioactive metabolite production [46,70,71], sponge cell culture would be an obvious method to use. *In vitro* culture of sponges as dissociated sponge cells or tissue would provide a clean and defined system for the production of sponge metabolites. However, attempts to develop continuously proliferating cell lines from sponges have failed so far. When achieving a continuously dividing sponge cell culture, cells can be grown in controlled bioreactors and controlling circumstances and stimulating production inside the cells will be much easier than in whole sponges. In addition, the use of these bioreactors makes scale up also easier (Figure 4). The difficulty with obtaining sponge cell lines is that cells do not continuously divide, because it is not known what sponge cells need in order to grow [47,72]. However, phytohemaglutin promoted cell division of sponge cells in culture [47]. Another problem encountered in these studies is getting the culture axenic, as sponges themselves are not axenic. Using undifferentiated cells may improve cell adjustment to the new environment. Thus far archeaocytes are the most promising cells in sponges to use for cell culture due to their pluripotency. However, using embryonic cells as starting material for cell culture may improve the chance in obtaining continuously dividing cells significantly [73].

There are a number of observations that showed that besides stimulating cell proliferation, also prevention of cell death may be important for the development of continuous cell lines. These observations are slow growth rates of sponges [56,60], in combination with fast healing of wounded tissue [63], reorganization of sponge tissue [74], DNA replication and no net growth of primmorphs [75] and fast DNA replication and probably apoptosis in whole sponge specimens [73]. We hypothesize that sponges may grow fast and die fast at the same time. This could be the reason that sponges are successful animals during evolution. Cells will not show any malfunctioning due to ageing because of the high turn over of cells, and the organism as a whole responds to attacks simply by giving up that part and building new cells. If this is true then sponges are very dynamic organisms that show a very slow net growth as a result of fast cell division and at the same time a high rate of apoptosis. For the development of continuous growing cell lines the strong capability of sponge cells to divide should be used and apoptosis should be prevented. A final advantage of sponge cell culture is that when understanding metabolic pathways and inducing factors for metabolite production the medium and culture conditions can be easily adjusted to improve production rates.

### 3.3. Symbiont Culture

If symbiotic micro-organisms inside the sponge are responsible for the production of the bioactive metabolites then of course culturing these symbionts would be the best option. Thus far few publication have focused on culturing symbionts, and first successes have been obtained [76,77]. To culture sponge-associated symbiont remains very difficult because of the difficulty in isolating and pure cultivation. Some microorganisms will not grow in pure culture, but can form colonies in the presence of other microorganisms [78]. Isolation of sponge-associated bacteria was best when using oligotrophic media [77]. Addition of sponge extract seems to have a positive effect on cultivability [76]. Isolated morphotypes from different sponge species were mostly affiliated to the *Alphaproteobacteria*, although also members of the genus *Bacillus* were found regularly. One alphaproteobacterium was found to dominate the culture which had microbial activity. Although weak and unstable, the cells lost activity during culture [77]. Thus, symbiotic bacteria cultivation did work, but keeping bioactivity is difficult. However, Kennedy *et al*. [79] showed that 50% of the isolated bacteria from *Haliclona simulans* possessed antimicrobial activity. Bioactive metabolite producing symbionts in culture are still very difficult to obtain. However, nowadays, more efforts are made towards development of sponge symbiont culture.

### 3.4. Genetic Modification

Metagenomics is the genetic analysis of a complex microbial mixture that can be used to analyze sponge microbial associations. Metagenomic approaches have also been used in which large gene fragments important for production of the bioactive compounds are identified, which could then be transferred to a suitable host [80]. This is a very promising technique, but a complex biosynthesis pathway means involvement of many genes, all of which need to be identified and all need to be transferred to the other host. Metabolic engineering is under development and the number of successes in introducing combinations of many genes is increasing lately [81–83].

For plant natural products recent advancements were made in the production of terpenoids, phenylpropanoids and alkaloids by using different techniques including protein engineering, codon optimization and combinatorial biosynthesis [84]. Paclitaxel (Taxol) is a classical example of a plant natural product produced in *Taxus brevifolia*. Taxol is a complex terpenoid that can be used for the treatment of breast cancer. Recently, a step forward was made in the production of Taxol in the yeast *Saccharomyces cerecisiae*. Heterologous genes were introduced encoding biosynthetic enzymes for the beginning of the taxoid biosynthetic pathway, as well as a regulatory factor inhibiting competitive pathways [82]. Especially the combination of combinatorial biosynthesis and elements to inhibit competitive pathways made it possible to increase the production of the intermediate taxadiene in yeast 40-fold to 8.7 mg/L.

The group of Keasling of the University of California, Berkeley has made major progress in expressing multiple genes in both yeast (*Saccharomyces cerevisiae*) and bacteria (*Escherichia coli)*. For example, genes encoding for the anti-malarial drug artemisinin, a sesquiterpene, have been expressed in *S. cerevisiae*. A single plasmid was used to express a combination of three plant genes and 84% of the plasmids were stable in the cells producing the intermediate amorphadiene, whereas poor plasmid stability was found in cells synthesizing artemisinic acid [83]. *E. coli* has also been employed in the production of the intermediate amorphadiene [81]. A simple cloning system for expressing the whole pathway enabled identification of rate limiting enzymes, which then could be over expressed to increase production seven-fold [81]. Thus, development of expressing multiple genes in host organisms is in full speed and when biosynthetic pathways are identified in sponges this road should be open for sponge drug development as well. However, difficulties are still to be overcome to be able to produce the toxic end products in host organisms, as the bioactive compounds can have toxic effects to the host organism itself.

## 4. Concluding Summary

For the development of sponge derived drugs still major breakthroughs are necessary. The best method to produce the different compounds depends on various factors. First focus of sponge derived drug development should be on why, when, how, and where the compound is produced. When being able to answer all these questions the best production method can be chosen. Then, focus should be put on development of the production method. Thus far the most promising method is whole sponge production in the sea. This is due to the fact that we do not understand the needs of the sponge for both growth and production. In the sea all elements are available to the sponge for survival, growth and metabolite production. For clean and defined systems it is better to develop cell culture methods, either sponge cell culture, symbiont culture or other host organisms. Before proper production methods for sponge metabolites are developed still a lot of research is necessary.
